# Smart Delivery Systems Responsive to Cathepsin B Activity for Cancer Treatment

**DOI:** 10.3390/pharmaceutics15071848

**Published:** 2023-06-29

**Authors:** Vera S. Egorova, Ekaterina P. Kolesova, Manu Lopus, Neng Yan, Alessandro Parodi, Andrey A. Zamyatnin

**Affiliations:** 1 Scientific Center for Translation Medicine, Sirius University of Science and Technology, Sochi 354340, Russia; egorova.vs@talantiuspeh.ru (V.S.E.); kolesova.ep@talantiuspeh.ru (E.P.K.); 2 School of Biological Sciences, UM-DAE Centre for Excellence in Basic Sciences, University of Mumbai Kalina Campus, Vidyanagari, Mumbai 400098, India; manu.lopus@cbs.ac.in; 3 School of Environmental Studies, China University of Geosciences, Wuhan 430074, China; yanneng@cug.edu.cn; 4 Institute of Molecular Medicine, Sechenov First Moscow State Medical University, Moscow 119991, Russia; 5 Belozersky Institute of Physico-Chemical Biology, Lomonosov Moscow State University, Moscow 119991, Russia; 6 Faculty of Bioengineering and Bioinformatics, Lomonosov Moscow State University, Moscow 119991, Russia

**Keywords:** cathepsin B, proteolytic activity, enzyme-induced cleavage, peptide linkers, responsive nanocarrier

## Abstract

Cathepsin B is a lysosomal cysteine protease, contributing to vital cellular homeostatic processes including protein turnover, macroautophagy of damaged organelles, antigen presentation, and in the extracellular space, it takes part in tissue remodeling, prohormone processing, and activation. However, aberrant overexpression of cathepsin B and its enzymatic activity is associated with different pathological conditions, including cancer. Cathepsin B overexpression in tumor tissues makes this enzyme an important target for smart delivery systems, responsive to the activity of this enzyme. The generation of technologies which therapeutic effect is activated as a result of cathepsin B cleavage provides an opportunity for tumor-targeted therapy and controlled drug release. In this review, we summarized different technologies designed to improve current cancer treatments responsive to the activity of this enzyme that were shown to play a key role in disease progression and response to the treatment.

## 1. Introduction

Cancer remains a major issue for public health and new therapeutic approaches are needed to tackle this disease. In recent decades, more insights about tumor biology were discovered and some of them resulted in potential therapeutic targets [1,2,3]. These efforts enabled the definition of the chemical, physical and biological characteristics of the diseased tissue and highlighted the differences with the healthy counterpart. New pharmaceutical “smart” approaches were designed to exploit cancer physical properties like a reduced pH of the tumor microenvironment or increased oxidative stress (gluthatione overexpression) to generate targeted systems that release a therapeutic payload in response to these characteristics. In parallel, the exploitation of biological abnormalities remains a preferential approach to targeting tumor cells and tissue. The occurrence of an abnormal proteolytic network represents a valuable target in tumor and other diseases and recent advances in biotechnology applied to drug delivery allowed to generate therapeutic tools responsive to proteolytic activity, characterizing inflammation [4,5], and in particular, cancer disease [6].

Within the large portfolio of proteases involved in tumor biology, lysosomal cathepsins were shown to participate in cancer development, spreading and promoting tumor survival, resistance to current treatments and migration [7]. Cathepsins is a family of 15 different proteases with redundant and overlapping activities. Among them, cathepsin B (CtsB) was particularly investigated in cancer, since its upregulation in various kinds of tumor [7,8,9,10,11,12]. CtsB overexpression in a tumor can be very prominent and even nearly 50 years ago, it was estimated that the mature active form of this enzyme could represent 20% of the total lysosomal proteases in cancer cells [13]. The increased CtsB activity inside and outside the cell in cancer suggested the development of therapeutic and diagnostic approaches based on CtsB activity targeting [14].

Currently, CtsB is the subject of numerous studies aimed at developing inhibitors to mitigate its activity in various conditions. The efficacy of CtsB inhibition has been tested in ameliorating a spinal cord injury by inhibiting macrophage ferroptosis [15] and treating cancer through the development of small molecules, such as the Nostoc-species-derived oxadiazine Nocuolin A [16], or biologics based on peptides [17,18]. The critical roles and regulatory pathways mediated by CtsB in various forms of regulated cell death, including pyroptosis, ferroptosis, necroptosis and apoptosis [19], make this enzyme highly attractive for developing new strategies based on inhibitory approaches. However, the significant expression of CtsB in cancer tissue also provides therapeutic avenues designed to leverage the proteolytic activity of this enzyme.

For example, pan-cathepsin diagnostic probes enabled tumor tissue imaging to increase the efficiency of surgical removal [20]. Different conjugates containing CtsB-cleavable linkers and nanoplatforms sensitive to CtsB proteolytic activity were developed to release the therapeutic payload as a function of CtsB activity. In this review, we summarized recent technologies applied to cancer disease that base their working mechanism on CtsB cleavage. However, before describing these therapeutic tools, a brief overview of CtsB activity in physiological conditions and tumor disease is necessary.

## 2. Cathepsin B Synthesis and Physiological Function

**Figure 1:**
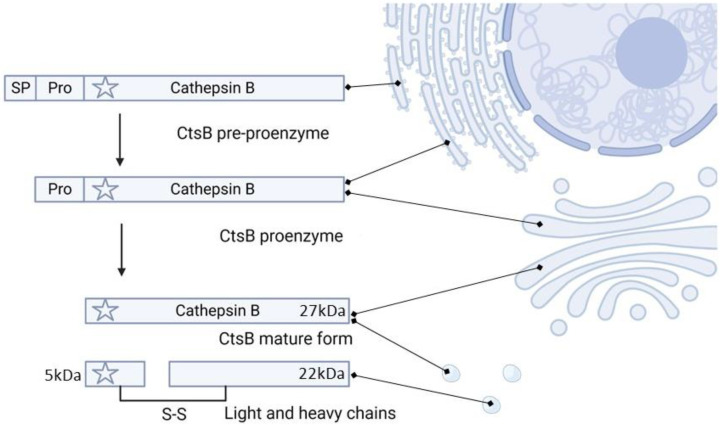
Schematic representation of cathepsin B maturation. Preprocathepsin B is synthesized into the rough endoplasmic reticulum. The cleavage of the signal peptide (SP) results in the formation of procathepsin B, which is transferred to the Golgi apparatus and finally to lysosomes (see the detailed description of the maturation process in the text). The star indicates Cys29 in the active site. The figure was generated using www.biorender.com, accessed on 17 March 2023.

CtsB is synthesized on the ribosomes of the rough endoplasmic reticulum as a 339 amino acid pre-proenzyme and then is processed in the N-terminal sequence to generate the proenzyme. The proenzyme is trafficked to the Golgi apparatus, where it is glycosylated with mannose-6-phosphate residues, and finally sorted to the endosomes and lysosomes [21,22]. In the acidic environment of the lysosomes, the proenzyme undergoes auto-processing to form enzymatically active mature CtsB (27 kDa) or double chain form (heavy chain (22 kDa) and light chain (5 kDa), linked with S-S bonds) (Figure 1) [23].

**Figure 2:**
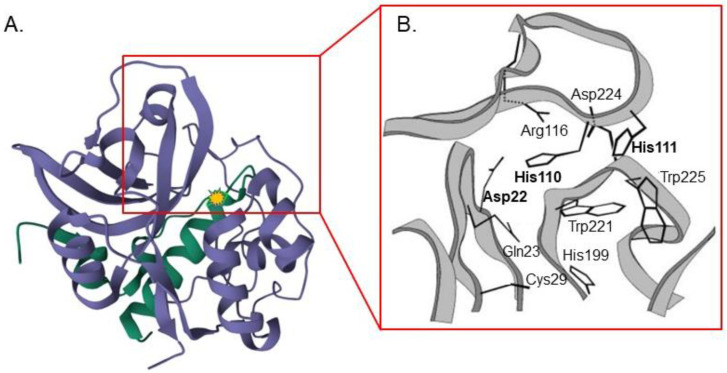
Structure of human cathepsin B. (**A**) Ribbon diagram of the enzyme. The heavy chain is shown in blue and the light chain is shown in green. The yellow mark indicates Cys29 residue in the active site (PDB ID: 1CSB, https://doi.org/10.2210/pdb1CSB/pdb) [29]. (**B**) Occluding loop of human cathepsin. (**B**) During the interaction with the substrate, Cys29 and His199 act as the catalytic nucleophile and general base. Gln23 stabilizes the oxyanion tetrahedral intermediate, whereas Trp221 and Trp225 form a hydrophobic pocket around the active site. Residues mediating the peptidyldipeptidase activity are indicated in bold. Ion pairs are formed between Asp22 and His110, and between Arg116 and Asp224, whereas His111 is unpaired. Figure reprinted from Krupa et al [26].

CtsB is a cysteine protease since this enzyme comprises a nucleophilic cysteine residue in the active site (Figure 2A) [24]. Its proteolytic activity involves three main stages: recognition/binding, cleavage and release. A delay in any of these steps might decrease the efficiency of the cleavage. The substrate allocation in the active site cleft is considered the most important stage of the proteolytic reaction since substrate cleavage and release depend on it [25]. CtsB activity results from the effective interaction between negatively charged C-terminal carboxylate groups and positively charged side chains His110 and His111 residues of the enzyme (Figure 2B) [26]. Unlike most other cathepsins, CtsB is characterized by both exo- and endopeptidase activities that depend on the microenvironment pH [22,27]. At acidic pH (ranging from 5.5 to 4.5), CtsB shows mostly exopeptidase properties, while at physiological pH values, endopeptidase activity is more prominent [28].

In normal conditions, CtsB is localized in the lysosomes, and autophagosomes [22] mediating cell turnover via macroautophagy of misfolded proteins and damaged organelles [30] antigen presentation in specialized immune cells [31]. In the lysosomes, CtsB shows maximal enzymatic activity, however, because of endogenous and/or exogenous triggers (i.e., reactive oxygen species or lysosomotropic compounds [32,33]), CtsB can translocate in the cytosol, in the nucleus or be secreted in the extracellular space, where it is involved in fundamental physiologic functions like wound healing, prohormone activation [4,34,35] and bone and neural tissue remodeling [36]. In the cytosol, CtsB can cleave the pro-apoptotic factor Bid, leading to cytochrome c release from mitochondria and, ultimately, to caspase-dependent apoptosis [37,38,39]. Alternatively, CtsB can also induce caspase-independent cell death as demonstrated after cell treatment with riccardin D [33]. This molecule induces lysosome membrane permeability resulting in CtsB translocation into the nucleus where it cleaves BRCA1 and mediates cell death by DNA damage accumulation. In the nuclei of senescent microglia cells, CtsB was shown to degrade sirtuins, including sirt1—nuclear deacylase, that protects the cells from oxidative stress and maintains chromatin stability [40], and to degrade mitochondrial transcription factor A; both processes are related to aging [38].

## 3. CtsB Activity and Targetability in Cancer

Despite its proven role in mediating cell death, CtsB activity was associated with cancer recurrence, metastasis, shorter survival and poor prognosis [8,9,10]. CtsB overexpression was demonstrated in different oncologic diseases, including breast [12,41], skin [42], colorectal [43], ovary [44], gastric [45] and renal cancer [11,42,46]. CtsB can promote tumor development in different ways: first, it is a crucial effector of autophagy supporting cancer cell growth and proliferation; second, increased CtsB activity in the tumor extracellular space (characterized by an acidic pH), results in the cleavage of the basal membrane components, including laminin, collagen V, collagen I, cell adhesion molecules (i.e., E-cadherin) [47,48] and cellular tight junctions. These activities can favor cancer cell motility, migration [42], epithelial-mesenchymal transition [48], invasion and angiogenesis, ultimately determining tumor development and metastasis. Upregulation of CtsB is tightly correlated with the activity of other proteases, which were shown to favor cancer progression, like the urokinase plasminogen activator (uPA)/plasminogen/plasmin [49], matrix metalloproteinase 9 [50] and overexpression of VEGF-C and TGF-β, resulting in angiogenesis promotion [51]. It was shown that CtsB downregulation alone or together with uPAR and MMP-9 using siRNA resulted in decreased growth, cell invasion and angiogenesis in glioma [52,53,54], malignant meningioma [55], prostate cancer [56] and reduced the incidence of bone and lung metastases in breast cancer [5,57]. These, and other pieces of evidence, make CtsB a prime target for the development of state-of-the-art smart delivery systems, which activate therapeutic effects in response to its proteolytic activity. This review primarily focuses on drug delivery platforms containing CtsB-cleavable peptide linkers. However, it is important to note that recently discovered CtsB-cleavable non-peptide linkers have also been incorporated into smart delivery systems [58,59]. In this scenario, nanocarriers releasing their therapeutic payload in response to CtsB activity represent a hot topic in pharmaceutical design. These systems can work both intracellularly after particle internalization and in the tumor microenvironment (TME), where CtsB can be secreted. The sensitivity to the enzyme eventually determines the delivery system’s therapeutic index, efficiency, pharmacokinetics and targeting. “Smart” carriers are supposed to respond quickly to the environmental variations of CtsB concentration and activity [60], while minimizing the payload release before reaching the target.

## 4. CtsB-Responsive Delivery Systems

### 4.1. CtsB-Sensitive Conjugates Based on GFLG and Other Peptides

CtsB-sensitive conjugates were developed to improve drug targeting and pharmacokinetics. Despite some works indicating that multidrug resistance (MDR) phenomena can arise against ADC [61], nanoformulations demonstrated effective cellular uptake and controlled intracellular payload release, which mitigate the MDR effects [62]. In this scenario, different multifunctional delivery systems were developed [63] based on CtsB-cleavable linkers.

GFLG (Gly-Phe-Leu-Gly) represents one of the most widely used tetra-amino acid linkers for developing CtsB-responsive drug delivery systems. The cleavage site of the GFLG linker is the amide bond between F and L, and after the C-terminal G [64]. This peptide was first used by Kopecek [65] to create drug formulations conjugating doxorubicin (DOX) and meso chlorin e6 mono (N-2-aminoethylamide). The use of this linker favored the release of the drug in the lysosomes and its further diffusion through the lysosomal membrane into the cytoplasm and, ultimately, into the cell nucleus. A drug conjugate of DOX bounded to the cell-penetrating peptide TAT using the GFLG linker peptide was synthesized to increase drug efficacy against liver carcinoma cells [66]. This peptide was used to connect covalently TAT to one, two or four DOX molecules and showed several advantages compared to the free drug, including overcoming MDR phenomena (according to Wenders’s strategy [67]) and increasing effective therapeutic concentration [68]. DOX release occurred exclusively via the cleavage of the GFLG linker, with no contribution from the spontaneous hydrolysis of peptides. The level of drug loading has been observed to have an inverse effect on drug release efficiency, while simultaneously increasing intracellular uptake. For instance, high degrees of drug conjugation could hinder CtsB access to the linkers because of steric influences, which is not the case in low degree conjugation. When compared to single molecule conjugation, modifications using 2 or 4 DOX molecules resulted in a decreased drug release rate, offering the capability to finely regulate drug release.

Over recent decades, many efforts were devoted to investigating systems based on the combination of GFLG peptide and HPMA (N-(2-hydroxypropyl) methacrylamide) polymer [69,70]. This technology was developed first in 1976 [71], and since then, many optimizations were performed. The HPMA impart high circulation time properties to the drug that can accumulate in the tumor through passive extravasation. In particular, CtsB-sensitive GFLG peptide was used to conjugate paclitaxel (PTX) and gemcitabine (GEM) to HPMA [72]. The pharmacokinetics and therapeutic efficiency of diblock, tetrablock and hexablock HPMA-PTX and –GEM conjugates were estimated in vivo against human ovarian carcinoma A2780 xenografts after intravenous and intraperitoneal administration. In all cases, the conjugation allowed for increased drug therapeutic effect and the best results were shown with simultaneous combinations of the payloads [72]. HPMA-GFLG conjugates were covalently bound also to DOX and investigated in clinical trials to treat lung carcinoma, colorectal cancer and anthracycline-resistant breast cancer [73]. The system showed a maximum tolerated dose of 320 mg/m^2^ with dose-limiting adverse effects comprising febrile neutropenia and mucositis. However, no significant side effects related to cardiac functions (typical of these drugs) were observed, despite individual cumulative doses up to 1680 mg/m^2^.

These peptides were also used to engineer dendrimers with improved delivery properties [74] including water-solubility, biodegradability and bio- and immune compatibility [75]. The payload could be covalently bound [76] or encapsulated inside [77] hyperbranched structures. To date, third-generation poly(amidoamine) (PAMAM) was modified by a tri-component ligand named PGM via maleimide active polyethylene glycol (mPEG) binding for dual pH/CtsB dependent DOX delivery [78]. The PGM ligand comprises the nuclear targeting sequence PKKKRKV, the CtsB-sensitive GFLG peptide and the pH-sensitive molecules morpholine. DOX was loaded into the PAMAM-PEG-PGM system in which morpholine charge switching favored lysosomal escape while the nuclear localization was mediated by importin α/β receptor targeting. In vitro studies showed a high efficiency of payload release because of the dual effect of CtsB and TME pH, while in vivo, the system showed a high inhibition efficacy against H22 mice tumor.

Bouilloux et al. [79] developed a polymeric prodrug releasing the photosensitizer Pheophorbide in the presence of high levels of CtsB. The system included several pheophorbide molecules attached to a poly-L-lysine backbone via the short CtsB-cleavable peptide GAGRRAAG. This design allowed for effective luminescence quenching of the photosensitizer and a loading of 28% of the lysine side chains. The CtsB-mediated cleavage of the peptide linker resulted in the photosensitizer’s release with consequent activation of the photoactivity properties. The cleavage of the prodrug was shown mainly in the bone marrow cells as a model of cancer cells with high proliferation activity and high level of CtsB expression and the peak of cellular uptake was registered 1 h after administration. After irradiation, a dose-dependent decrease in cell viability was observed because of ROS generation, while no toxicity was detected in the absence of irradiation. Ex vivo studies confirmed the safety of this system, with no adverse effects on the integrity of the vessels. The observed photodynamic therapy (PDT) effect was significantly reduced by pre-treating the cells with E64d cysteine protease inhibitor affecting CtsB activity, demonstrating the specificity of this approach [80].

Systems based on reactive drug molecules were developed as well. In particular, GEM was modified via a maleimide based reaction with a CtsB-sensitive linker. This complex was designed to link covalently circulating serum albumin via an amide bond (binding efficiency of 90%, 1 min after the administration) [81]. The system was shown to be stable at physiological pH, while at pH 5.4, drug release was induced in the presence of CtsB. The efficacy of this system was shown against CtsB-expressing 4T1 breast cancer cells compared with other cancer models expressing this enzyme at lower levels.

### 4.2. Antibody-Drug Conjugates

**Figure 3:**
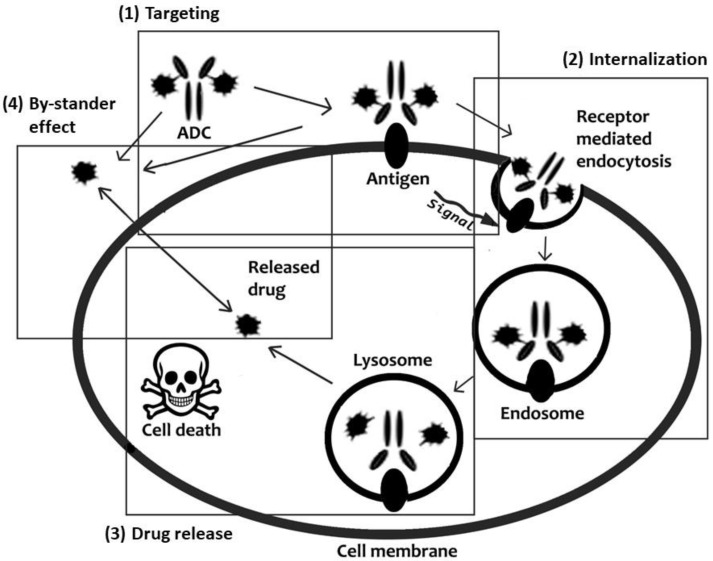
Schematic representation of ADC mechanism of action: targeted drug delivery and release. Figure reprinted from Ponziani et al. [83].

Antibody-drug conjugates (ADC) have been investigated since the early-1980s [82] for their ability to deliver a therapeutic to a specific target and they represent one of the most solid approaches to improving the pharmacokinetics properties of toxic drugs. Their working mechanism is based on the identification of surface biomarkers that are overexpressed in abnormal cells, representing the target against which the antibody specificity is addressed. ADC technology is composed of three elements comprising the antibody, the drug and a linker connecting these two components [83]. The linker design enables drug targeting via antibody binding and, moreover, determines PK properties of the ADC. This phenomenon is very important to improve the therapeutic properties of drugs affected by fast clearance. Current research in the field is focused on generating antibodies with higher specificity and biocompatibility and improving current synthesis techniques to conjugate multiple therapeutics to the antibodies. However, much effort is also dedicated to developing degradable linkers to favor and control drug release. The first goal of the linker is to guarantee product stability in circulation. However, after reaching the target, the therapeutic molecule has to be released to avoid the unwanted accumulation of the curative payload in not optimal subcellular compartments [84]. In this context, it is important to highlight that if the drug is not released, it will accumulate in proximity to the cell membrane or, in the case of antibody receptor-mediated endocytosis, in the lysosomes. ADC characterized by non-degradable linkers rely on the degradation of the whole antibody to allow drug diffusion in the cytoplasm, but charged residues connected to the payload may hamper this phenomenon [85]. Lysosomal proteases represent an optimal trigger for drug release, and for this reason, the development of linkers sensitive to CtsB activity was extremely investigated. As extensively reported above, CtsB is overexpressed in many cancer cells, representing an additional mechanism of targeting. In addition, this protease can be secreted in the extracellular space favoring the release of the drug molecule, also from the non-internalized antibodies (Figure 3). This phenomenon is known as the bystander effect, and it results in killing cancer cells that reside in proximity to the targeted cells but that do not overexpress the target surface biomarker. The investigation of CtsB sensitive linkers highlighted the opportunity to use different dipeptides that are currently used to generate ADC [86]. The most common sequences are represented by Phe-Arg (FR) [87], Val-Cit (VC), Phe-Lys (FK) and Val-Ala (VA) [83]. Fu et al. [88] exploited the VA linker to generate new treatments against hepatocellular carcinoma by combining Duocarmycin SA and pyrrolobenzodiazepine (PBD) dimer to antibodies targeting GPC3 on hepatocellular carcinoma cell membrane. These linkers are usually modified at one or both of their terminals with chemical groups with structural functions to favor the attachment of the peptide to the antibody or to the drug and increase the enzymatic access to the peptide. One of the most common chemical modifications in this field is represented by the para-aminobenzyloxycarbonyl (PABC) group, that showed self-cleavage properties (self-immolative spacer) after CtsB hydrolyzation of the peptide linker [89].

Different ADC were FDA-approved for cancer treatment and many of them use CtsB-sensitive linkers. Enhertu is an ADC composed of Transtuzumab (directed against tumor cells over-expressing Her-2) and was approved to treat breast [90], gastric [91] and non-small cell lung cancer [92]. The antibody is conjugated via the pentapeptide maleimide GGGPG (Gly-Gly-Gly-Pro-Gly) to the exatecan derivative Dxd. This linker is sensitive to CtsB and CtsL activity and is fundamental to control the release of Dxd where these proteases are overexpressed. Zynlonta comprises an antibody specific for CD19 conjugated via a VA linker to the DNA alkylating agent SG3199 and it was approved for treating B-cell non-Hodgkin lymphoma, over-expressing CD19. The ADC Adcetris was approved to treat Hodgkin lymphoma and systemic anaplastic large cell lymphoma and it is based on an antibody directed against CD30 (also known as the Reed–Sternberg cell-associated antigen). This antibody is conjugated to the drug monomethyl auristatin E (MMAE), through two chemical spacers (maleimidocaproyl and PABC groups), separated by the CtsB-sensitive VC dipeptide. The spacers have the primary function of physically conjugating the antibody, the linker and the MMAE, but also to favor the access of the enzyme to the peptide, that could be eventually affected by the drug [85]. This very similar technology was exploited to design the FDA-approved ADC Polivy and Padcev delivering MMAE. Polivy is used to treat diffuse large B-cell lymphoma by targeting CD79b while Padcev was approved to treat advanced or metastatic urothelial cancer and is composed of an antibody directed against Nectin-4 [93]. Tivdak comprised a human anti-TFIgG1κ antibody conjugated to MMAE via the CtsB-cleavable maleimidocaproyl-VC-PABC linker and was FDA-approved in 2021 to treat adult patients with recurrent or metastatic cervical cancer [94]. Disitamab vedotin, also known as RC48, with the same technology and CtsB sensitive linker, was approved by NMPA (China) to treat metastatic gastric cancer [95].

Interestingly, a deeper investigation of this linker showed that CtsB expression inhibition through different biomolecular techniques did not affect the cytostatic properties of the system. This phenomenon was due to compensating mechanisms originating from the activity of other proteases (i.e., CtsS) mitigating the lack of CtsB. It is also worth highlighting that linking MMAE via a non-cleavable enantiomer did not completely suppress the cytostatic properties of the system because a toxic catabolic product of MMAE can generate in the lysosomal compartment. On the other hand, the cytotoxic dependence on CtsB activity was restored by substituting MMAE with another drug (pyrrolo [2,1-c][1,4]benzodiazepine dimer) that did not generate any toxic catabolite in the lysosomes. This work is important because it showed that functional ADC can be generated despite the linker sensitivity for a specific protease [96].

A different category of ADC agents has been developed utilizing PBD dimers, which are roughly 50–100 times more potent than the standard drugs used in the creation of ADCs (such as MMAE). Two examples of such agents are SGN-CD33A (Vadastuximab talirine) [97] and SGN-CD70A [98], which both include the same talirine cleavable linker, a maleimidocaproyl linker with a CtsB-sensitive VA-PAB moiety, PBD dimer (SG 1882) and anti-CD33 and anti-CD70 antibodies, respectively. Unfortunately, the clinical trials for these drugs had to be terminated due to severe side effects and increased patient mortality [99]. Furthermore, SGN-CD123A, ADC with a similar structure based on an anti-CD213 antibody, was developed to treat acute myeloid leukaemia [100].

Another ADC drugs containing CtsB-cleavable tesirine linker (tesirine linker in its turn comprises Val-Ala peptide) are evaluated in the ongoing clinical trials: Rovalpituzumab tesirine is an anti-DLL3 ADC developed to treat small cell lung cancer (phase III) and loncastuximab tesirine [101] and camidanlumab tesirine [102] are anti-CD19 and anti-CD25 ADCs (phase II), respectively, indicated to treat B cell acute lymphoblastic leukemia and Hodgkin lymphoma.

In a pre-clinical work, it was discovered that Carboxylesterase 1C can break down VC linkers in the plasma [103] affecting the complex stability in the blood. Nevertheless, modifying the linker by introducing an aminocaproyl or additional Glu residue upstream of the VC dipeptide has been shown to mitigate this issue [93,104], while maintaining or increasing sensitivity to CtsB activity.

The most common CtsB-sensitive peptide linkers and their basic physicochemical properties are summarized in Table 1.

### 4.3. CtsB-Sensitive Nanoparticles

Nanocarriers were shown to improve drug delivery owing to their unique properties such as small size, large specific surface area and targeting surface modifications [105,106]. In addition, carrier physicochemical properties can be tuned by changing their composition, size, shape and surface properties [107]. In this scenario, the cetuximab–VC–DOX targeting tumor cells overexpressing epidermal growth factor receptor (EGFR) was adsorbed on BSA nanoparticles (NPs) [108] synthesized via desolvation [109]. A viability assay performed in EGFR-overexpressing RKO cells showed stronger cytotoxicity of cetuximab-VC-DOX-NPs in comparison with similar systems generated with an unspecific antibody. The VC peptide linker was shown to increase the circulation time of the drug and favor the release of the payload because of CtsB activity. A folate receptor (FR)-targeting liposome encapsulating a PTX-dendrimer conjugate was generated to provide enhanced targeting properties against breast cancer cells overexpressing FR and CtsB [110]. The PTX molecules were linked to the dendrimers via the CtsB-sensitive GFLG peptide linker [111]. Compared to traditional FR-targeting liposomes, this system showed higher prodrug retention, increased cytotoxicity to cancer cells and more effective tumor shrinking in vivo.

**Figure 4:**
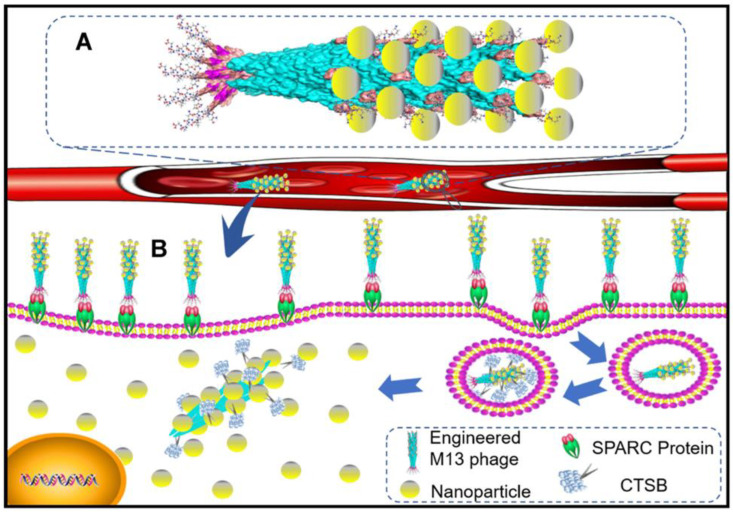
Schematic diagram of CtsB-sensitive M13 phage-SPION system: (**A**) Schematic diagram of the system. (**B**) Mechanism of payload delivery and release. Figure reprinted from the International Journal of Nanomedicine 2021 16 7091–7102 [113]. Originally published by and used with permission from Dove Medical Press Ltd.

Superparamagnetic iron oxide NPs (SPION) were complexed with a genetically modified M13 phage [112] (Figure 4) to target MDA-MB-231 human breast cancer cells [113]. The M13 phage co-expressed SPARC binding peptide to target cancer cells and the CtsB sensitive peptide DFK to bind the SPION. This corn-like structure demonstrated great cellular uptake and lysosomal sequestration. The CtsB cleavage of DFK linkers promoted SPION release in the target cells, allowing hyperthermia-based treatment. Interestingly, when administered to the cells, the system could induce CtsB mRNA expression.

Ehrsam et al. [114] generated poly(dimethylsiloxane)-b-poly(methyloxazoline) (PDMS-PMOXA) NPs modified on their surface with the CtsB-responsive Fmoc-aminocaproic acid(Ahx)-GSGFLGSC peptide bearing PTX. A significant increase in OVCAR-3 and OVCAR-5 cell lines’ cytotoxicity was observed when the particle treatment was combined with purified CtsB, indicating that the enzyme could accelerate the release of the payload, while the addition of the CtsB inhibitor CA-074 decreased particle toxicity [115].

For cancer treatment, rare-earth doped upconversion nanocrystals (UCN) were embedded in a polymeric matrix bearing the photosensitizer Chlorin e6 (Ce6) and modified with the CtsB-sensitive peptide Ac-FKC(StBu)AC(SH)-CBT [116]. The peptide contained a protected reactive group comprising 2-cyanobenzothiazole that could covalently bind cysteine. CtsB cleavage of the peptide favored the covalent cross-linking between the exposed cysteine and 2-cyanobenzothiazole of adjacent particles, ultimately favoring their aggregation. UCN cross-linking enhanced their upconversion emission properties upon laser irradiation (wavelength of 808 nm) and the consequent generation of singlet oxygen by Ce6. The aggregation of UCN in an environment with a high content of CtsB was confirmed by electron microscopy and was accompanied by a shift in the UCN absorption peak. In vitro and in vivo fluorescence and photoacoustic imaging studies confirmed the success of this enzyme-induced cross-linking reaction.

Recently, self-assembling prodrug NPs have been proposed to generate a new concept of nanodelivery systems [117]. NPs assemble could occur by modifying the payload with hydrophobic or amphiphilic linkers inducing a particle self-assembly process. The prodrug molecules were modified with ester, thioether, thioketal and disulfide groups allowing for a selective activation of the system in the TME [118]. Proteolytic sensitivity against a particular enzyme could be imparted using sensitive peptides in the formula. It was known that Phe was the key amino acid residue for creating self-assembling nanostructures because of intermolecular hydrophobic and π-π interactions [119]. Peptides with the CtsB-cleavable sequence FFKF (Phe-Phe-Lys-Phe) demonstrated effective nanofiber formation and DOX loading and release efficacy [120]. The FRRL-DOX (Phe-Arg-Arg-Leu-DOX) self-assembled NPs (170 nm) showed a 16-times higher tumor targeting than free DOX [121].

Over the last year, only a few works have been published about CtsB-sensitive nanoparticles. One such work, by Huang et al. [122], involved the development of ferumoxytol nanoparticles that were linked to the MMAE drug by four polyethylene glycol linkers, a CtsB-cleavable VC dipeptide and a p-aminobenzylcarbamate spacer. These nanoparticles were tested in vitro using the U87-MG glioblastoma cell line and in vivo on nude mice that had been injected intracranially with these cells. The resulting nanoparticles were found to be effective in inducing the death of glioblastoma cells. The maximal anticancer effect was achieved through a combination of nanoparticle treatment and radiotherapy. Another work by Shi et al. [123] aimed to develop a complex of fluorocarbons linked with polyarginine and CtsB-sensitive GFLG peptides to anti-VEGF siRNA. Positively charged nanoparticles, about 90 nm in size, were prepared and the polyarginine and GFLG peptide sequences provided double cleavage mediated by GSH reduction and CtsB activity. The work resulted in efficient siRNA release and VEGF deregulation in HeLa cervical carcinoma cell line.

## 5. CtsB-Cleavable Surface Modifications

Most surface functionalized ”gated” stimuli-sensitive systems comprise porous carriers whose loaded pores are sealed with biodegradable “gatekeepers”, designed to dissolve upon specific external or TME triggers (i.e., pH, temperature, redox potential, light and enzymatic activity) [124]. Within these technologies, mesoporous silica nanoparticles (MSN) were investigated for the accommodation and controlled release of drugs [125,126,127].

This is the case of drug-loaded MSN [128], in which the surface was functionalized with alkoxysilane tether, α-cyclodextrin, the CtsB-cleavable peptide GFLG, arginine-rich cell-penetrating peptides (R7) and the tumor-targeting peptide RGD (targeting αvβ3 integrin). The authors showed that in cells with a different CtsB expression (normal and cancer cells), the enzyme could induce a burst release of the payload and an increased cytostatic activity. A similar technology was developed to deliver safranin O or DOX exploiting the CtsB-sensitive capping peptide alkinil-GIVRAKEAEGIVRAK-OH (P) [129]. This system has been tested on multiple cell lines with different CtsB expression levels in combination with specific CtsB inhibitors. A significant cytotoxic effect was observed only in the cells with a high CtsB expression, while the specific inhibitors contrasted the anticancer activity of the system.

**Figure 5:**
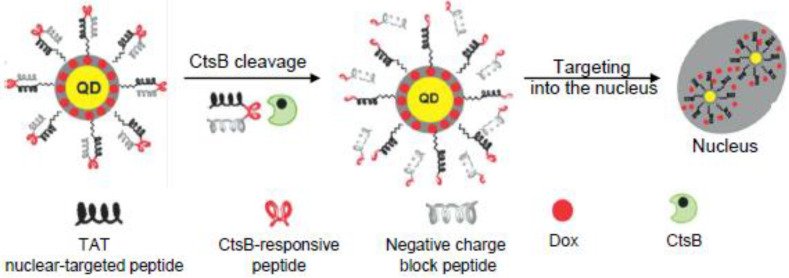
Schematic illustration of the CtsB-sensitive nuclear-targeted QD@mSiO_2_ nanoparticles. Figure reprinted from Li et al. [130]. Originally published by and used with permission from John Wiley & Sons, Inc.

In another report, a similar system for nuclear targeting was proposed. Quantum dots (QD) were coated with a shell of mesoporous silica [130] loaded with DOX. In this technology, the QD allowed for continuous real-time monitoring of the nanocarrier trafficking. The surface of the particles was modified with three peptides: (i) a nuclear-targeted oligocationic TAT peptide; (ii) the short enzyme-cleavable peptide linker PGFK closing the pores; and (iii) an anionic-inhibitory domain to neutralize the positive charges of the other peptides (Figure 5). The nanocarriers were stable and inactive in the absence of CtsB. However, after tumor cell internalization, the proteolytic activity of CtsB destabilized the tri-peptidic system and activated the TAT residues on the QDs@mSiO_2_ surface that favored DOX nuclear targeting and consequent tumor cytotoxicity, especially in the cells characterized by drug-resistant properties.

Recently, gold nanorods (AuNRs) were chosen as the delivery platform to accommodate a drug formulation named LAX [131]. LAX is composed of three components: (i) GFLG tetra-peptide sensitive to CtsB; (ii) DOX; and (iii) lipoid acid (LA) which allowed for the formation of a DOX coating on the particle surface via SH group. The GFLG peptide increased the stability of the system and after AuNR–LAX internalization into cancer cells, CtsB-mediated cleavage induced DOX release. Furthermore, AuNRs showed local surface plasmon resonance properties detectable in the NIR region. DOX release was monitored by detecting free drug fluorescence, since auto-quenching phenomena occurred when the drug was stabilized on the particle surface, while AuNR could be exploited for photothermal therapy. The system was tested on a human breast cancer cell line showing a high potential of AuNR–LAX in overcoming multi-drug resistance.

Gotov et al. [132] synthesized AuNPs modified with hyaluronic acid for targeted delivery of docetaxel (DTX). The hyaluronic acid was attached to the surface of the AuNPs using the CtsB-sensitive peptide GFLGC and allowed for increased circulation and targeting properties against the CD44 receptors overexpressed on cancer cells. The system showed greater cytotoxicity and higher tumor suppression efficacy in vivo than free DTX, providing a means to combine thermoablation and chemotherapy.

In a recent study conducted by Li et al. [133], it was demonstrated that resveratrol encapsulated in mesoporous silica nanoparticles linked to transferrin molecules with CtsB-cleavable DEGFLGED peptide, exhibited high anticancer properties. In this case, transferrin acted as a capping and targeting agent. The resulting nanoparticles effectively reduced the viability of MCF7 cells and increased the apoptosis rate up to 80.8%. The authors have also planned to conduct further research using a mouse xenograft model.

The aforementioned CtsB sensitive drug delivery systems are summarized in Table 2.

## 6. Conclusions

In this review, we have highlighted the potential of developing CtsB-cleavable technologies for targeting cancer cells and tumor microenvironments. Although ADCs have shown superior results in terms of translational purposes, the use of nanosystems may expand the portfolio of possible therapies, such as photodynamic and photothermal therapy. However, although CtsB-cleavable conjugates, nanoparticles, ADCs and surface modifications are extensively studied in preclinical and even clinical settings, more work is necessary to define the specificity of these systems, since cathepsin proteases are numerous and have redundant activity. Additionally, the malignant properties of cancer cells must be identified as a function of CtsB overexpression, even though this enzyme is ubiquitous and expressed at varying levels in all cancer cells. Finally, for future clinical translation purposes, these systems need to be simplified in their synthesis as they often consist of various components. Fine control of the manufacturing process may be challenging, particularly for large-scale production.

## Figures and Tables

**Table 1:** Chemical structures and basic physicochemical properties of CtsB-cleavable linkers *.

Peptide Linker	Chemical Structure	Molecular Weight	Net Charge	Isoelectrical Point
GFLG	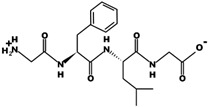	392.2	0	5.60
GAGRRAAG	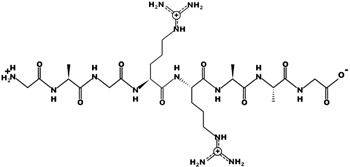	714.4	+2	12.49
VC	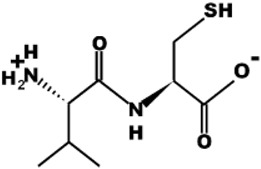	220.1	0	4.95
DFK	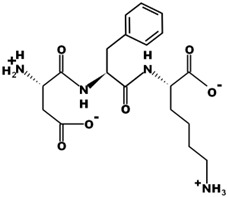	408.2	0	6.77
VA	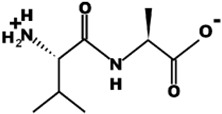	188.1	0	5.60
FFKF	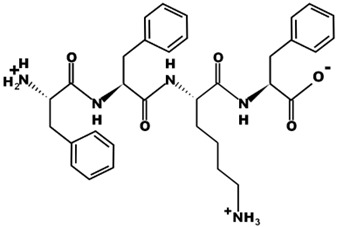	587.3	+1	9.93
PGFK	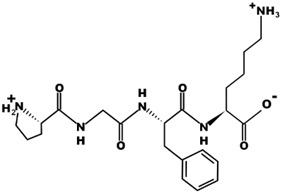	447.2	+1	10.59
GIVRAK	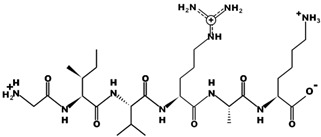	642.4	+2	11.56

* Based on data from www.pepdraw.com, accessed on 15 June 2023.

**Table 2:** The different systems for cathepsin B sensitive drug delivery.

Peptide Linker	Drug	Delivery System	Cancer	Outcomes	Ref.
GFLG	Doxorubicin	Conjugate	HepG2 cells	The conjugate structure had an opposite effect on DOX release and tumor accumulation. The synergistic effect of these properties exhibited the highest antitumor efficacy	[66]
GFLG	Paclitaxel and gemcitabine	Conjugate with HPMA dendrimers	A2780 human ovarian carcinoma cells	The combination of PTX, GEM and diblock structures yielded the highest inhibition efficacy of tumor growth	[72]
GFLG	Doxorubicin	Conjugate with polymer	Lung carcinoma, colorectal cancer and anthracycline-resistant breast cancer	Antitumor activity in refractory cancers was demonstrated, and polymer-drug conjugation has been shown to reduce DOX dose-limiting toxicity	[73]
GFLG	Doxorubicin	Conjugate with polymer	H22 mice tumor	The conjugates were successfully internalized into the cell nuclei, resulting in an inhibition efficiency of ~90% for the tumor	[78]
GAGRRAAG	Pheophorbide	Conjugate	Bone marrow cells	The photodynamic effect was demonstrated to be greater than 60%, and the system could be used as a sensor for cathepsin activity	[79]
VC	Doxorubicin	Nanoparticle	RKO colon carcinoma cells	Conjugates can efficiently bind to and be internalized by EGFR-overexpressing cancer cells. This strategy could be used to reduce systemic toxicity	[108]
DFK	SPION	Nanoparticle	MDA-MB-231 breast cancer cells	The increased efficiency of NP internalization and spion release following exposure to CtsB were demonstrated	[113]
GSGFLGSC	PTX	Nanoparticle	OVCAR-3 adenocarcinoma cells and OVCAR-5 ovarian cancer cells	The time-dependent PTX release and a 25-fold reduction in IC50 compared to pure PTX were demonstrated	[114]
Ac-FKC(StBu)AC(SH)-CBT	Chlorin e6	Nanoparticle	H-29 human colorectal adenicarcinoma cells	CtsB induced NPs self-assembly, resulting in an increased singlet oxygen generation and a significant enhancement of the photodynamic effect	[116]
FFKF	Doxorubicin	Self-assembled nanoparticle	Tumor lysates	A library of FFKF peptides with various N-terminal capping groups was studied, and their self-assembly and sensitivity to cathepsin B and L were analyzed. Cbz-FFKF-OH showed the highest potential and a release of 92% of DOX within 8 h	[120]
VA	Duocarmycin and pyrrolobenzodiazepine	ADC	Hepatocellular carcinoma	Using dipeptides, VC and VA, conjugating Duocarmycin SA and PBD dimers to antibodies targeting GPC3 on hepatocellular carcinoma cells advances in liver cancer therapy were achieved	[88]
VC	(Pyrrolo [2,1-c][1,4]benzodiazepine dimer)	ADC	BT474 carcinoma cells, KPL-4 breast cancer cells and BJAM lymphoma cells	The targeting agent used is of more importance for the effectiveness of ADC than the efficiency of linker cleavage	[96]
VC	Auristatin-based	ADC	Expi293 cells	Carboxylesterase 1C was identified as the enzyme responsible for the plasmatic hydrolysis of (VC-PABC)-based linkers	[103]
VC	Monomethyl auristatin E	Nanoparticle	U87 glioblastoma cells	The system provided efficient cellular uptake and high toxic effect on glioblastoma cells. The maximal anticancer effect was achieved using NPs and radiotherapy	[122]
GFLG	Anti-VEGF siRNA	Nanoparticle	HeLa cells	Efficient siRNA release and VEGF deregulation in HeLa cells were achieved	[123]
GFLG	Doxorubicin	Functionalized nanoparticle	HeLa cells	80% of DOX release was observed in 24 h in the presence of CtsB	[128]
GIVRAKEAEGIVRAK	Safranin O or DOX	Functionalized nanoparticle	Hela cells	A 5-fold increase in the release of Safarin O was observed in the presence of lysosomal extract, leading to a CtsB-dependent cytotoxic effect	[129]
PGFK	Doxorubicin	Functionalized nanoparticle	A549 human non-small cell lung cancer cells, NIH-3T3 mouse fibroblast cells, A2780 human ovarian cancer cells	At acidic pH, CtsB led to a four-fold increase in DOX release and consequent higher toxicity	[130]
GFLG	Doxorubicin	Functionalized nanoparticle	MCF-7 human breast cancer cell	The nanoparticles represented a promising system to overcome MDR phenomena	[131]
GFLGC	Docetaxel	Functionalized nanoparticle	HeLa and MCF-7 breast cells	The systems showed higher circulation properties, efficacy and safety	[132]
DEGFLGED	Resveratrol	Functionalized nanoparticle	MCF-7 breast cells	Anticancer activity exceeded 80%	[133]
VA	SG3199	ADC	B-cell non-Hodgkin Lymphoma	Zynlonta^®^ is FDA-approved ADC for the treatment of large B-cell lymphoma (USA)	[134,135]
VC	Monomethyl auristatin E	ADC	Hodgkin lymphoma	Adcetris^®^ was approved ADC for the treatment of Hodgkin lymphoma (USA)	[136,137]
VC	Monomethyl auristatin E	ADC	Large B-cell lymphoma	Polivy^®^ was approved for the treatment of large B-cell lymphoma (USA)	[138]
VC	Monomethyl auristatin E	ADC	Metastatic urothelial cancer	Padcev^®^ was approved for the treatment of metastatic urothelial cancer (USA)	[139]
VC	Monomethyl auristatin E	ADC	Metastatic cervical cancer	Tivdak^®^ was approved for the treatment of metastatic cervical cancer (USA)	[94]
VC	Monomethyl auristatin E	ADC	HER-2 positive solid tumors	RC-48^®^ was approved for the treatment of metastatic cervical cancer (China)	[95]
VA	SGD-1882	ADC	Positive acute myeloid leukemia	Clinical trials were stopped because of severe adverse events and increased patient mortality	[97]
VA	SGD-1882	ADC	Non-Hodgkin Lymphoma and Renal Cell Carcinoma	Clinical trials were stopped because of severe adverse events and increased patient mortality	[98]
VA	SG3199	ADC	Large B-cell lymphoma	Phase 2 of clinical trials of ADCT-402 (Loncastuximab Tesirine), NCT05296070 NCT05249959	[101]
VA	SG3199	ADC	Hodgkin lymphoma	Phase 2 of clinical trials of Camidanlumab tesirine NCT04052997	[102]

## Data Availability

Not applicable.
